# Effects of pre-training using serious game technology on CPR performance – an exploratory quasi-experimental transfer study

**DOI:** 10.1186/1757-7241-20-79

**Published:** 2012-12-06

**Authors:** Johan Creutzfeldt, Leif Hedman, Li Felländer-Tsai

**Affiliations:** 1 Department of Clinical Science, Intervention and Technology, Karolinska Institutet, K32, Stockholm, 141 86, Sweden; 2 Center for Advanced Medical Simulation and Training, Karolinska Institutet and Karolinska University Hospital, Stockholm, Sweden; 3 Department of Psychology, Umeå University, Umeå, Sweden

**Keywords:** Assessment, Avatars, Cardiopulmonary resuscitation, Educational technology, e-learning, MVW, Virtual learning environments, Patient simulation, Students, Young adults

## Abstract

**Background:**

Multiplayer virtual world (MVW) technology creates opportunities to practice medical procedures and team interactions using serious game software. This study aims to explore medical students’ retention of knowledge and skills as well as their proficiency gain after pre-training using a MVW with avatars for cardio-pulmonary resuscitation (CPR) team training.

**Methods:**

Three groups of pre-clinical medical students, n = 30, were assessed and further trained using a high fidelity full-scale medical simulator: Two groups were pre-trained 6 and 18 months before assessment. A reference control group consisting of matched peers had no MVW pre-training. The groups consisted of 8, 12 and 10 subjects, respectively. The session started and ended with assessment scenarios, with 3 training scenarios in between. All scenarios were video-recorded for analysis of CPR performance.

**Results:**

The 6 months group displayed greater CPR-related knowledge than the control group, 93 (±11)% compared to 65 (±28)% (p < 0.05), the 18 months group scored in between (73 (±23)%).

At start the pre-trained groups adhered better to guidelines than the control group; mean violations 0.2 (±0.5), 1.5 (±1.0) and 4.5 (±1.0) for the 6 months, 18 months and control group respectively. Likewise, in the 6 months group no chest compression cycles were delivered at incorrect frequencies whereas 54 (±44)% in the control group (p < 0.05) and 44 (±49)% in 18 months group where incorrectly paced; differences that disappeared during training.

**Conclusions:**

This study supports the beneficial effects of MVW-CPR team training with avatars as a method for pre-training, or repetitive training, on CPR-skills among medical students.

## Background

A major bottleneck for better outcome after cardiac arrest is the availability of trained layman rescuers. In Sweden 68% of witnessed out-of-hospital victims suffering sudden cardiac arrest are exposed to cardiopulmonary resuscitation (CPR) attempts by laymen rescuers [1,2]. In the US, the corresponding number is 44%, but with large variations between different areas [3]. Currently CPR training normally rests on a traditional model consisting of a theoretical introduction and individual manikin based procedural training. Although this type of training has evolved to the current state over the years, essentially the model itself has been unchanged. Also, in the current CPR guidelines, teamwork issues have impacted the recommendations [4-7], although clarifications of what characterizes effective teamwork during CPR are lacking [8].

With increasing computer literacy the use of computer gaming technology for learning and training, i.e. serious games, has been reported in several areas including medicine [9-17]. Theoretical benefits with this technology include the availability in remote settings and at free hours, but also inherent positive properties of the computer game technology in itself, e.g. the opportunity to tailor it to certain contextual demands and a property to match peoples’ level of knowledge and skills. Further, this technology enables experiential learning often with ample feed-back, creates a high level of engagement among participants, and carries the ability to switch context in order to support transfer [18-20].

By using multiplayer virtual world (MVW) technology with avatars it is possible to interactively practice situations involving several subjects. Although some positive results exist [21-23], to date there is a lack of knowledge on how effective serious games are in different training situations. Also, in general, it is believed that the effectiveness of training is dependent on many other factors than which modality is being used [24,25].

We have previously developed a MVW-CPR team training model (a serious game using avatars) and reported of its use among medical students. In a test-retest study we found that this training was feasible, popular and increased the subjects’ concentration as well as self-efficacy beliefs indicating that the participants experienced a higher level of preparedness [26]. Similar results were also seen in an international study on high-school students [27].

The aim of the current study was to explore if medical students pre-trained with MVW-CPR in teams had retained knowledge and skills and were able to transfer their proficiency gain to a full-scale simulator environment. Signs of transfer would support the value of using MVW technology for training CPR. Our main hypothesis was that subjects who had pre-trained using MVW would perform CPR faster and better in line with the guidelines. We also hypothesized that signs of retention would be greater proximal to training, and that retention would be greater for knowledge and skills that were actively trained as opposed to just lectured [28].

## Methods

### Recruitment and sample

This study was designed as an exploratory quasi-experimental controlled study with 3 groups: Two groups had attended MVW-CPR training, 18 and 6 months respectively, before the study (18 m and 6 m), and one group served as reference group (control). The latter group was matched to the other groups, consisting of peers attending the same semester of medical school. Thirty-six medical students at Karolinska Institutet volunteered to participate during their preclinical period (second and third year). All of them had attended a compulsory conventional manikin based CPR training course during their first semester of medical school. Subjects were enrolled by announcements. The study was approved by the regional ethics committee at Karolinska Institutet, and informed consent was obtained from the participants.

### MVW-CPR team training

The first phase of the study consisted of MVW-CPR team training. The training comprised two sessions. The first session started with a 10 minute rehearsal lecture on basic life support (BLS), followed by an approximately 20 minute long familiarization to the virtual environment. During this the participants learned how to control the avatar using the keyboard, communicate with each other using a headset with microphone, and performing various tasks. The actual team MVW-CPR training consisted of 4 short (4-5 minutes) scenarios. In these the subjects in groups of 3 had to take care of a cardiac arrest victim that collapsed in front of them. This involved approaching the victim, examining the victim and starting resuscitation as stated by the guidelines. The latter included an emergency phone call to the 911 dispatcher. The actions had to be performed in collaboration within the group. Following each scenario a brief (3-5 minute) feed-back session followed. Standard personal computers were used which were connected to the virtual world by broad band internet connections. After 6 months the subjects attended a similar session without the lecture.

### Loss of subjects

All subjects from previous MVW-CPR training agreed to participate, but due to personal competing interests some subjects could not attend during the short time frame the study had to be carried out in. In the 18 months group 4 out of 12 subjects were lost and in the control-group 2 subjects were lost due to scheduling difficulties before the start of this study. No subjects dropped out during the study in any group. Background data is shown in Table 1.

**Table 1:** Demographic data

**Subjects’ Characteristics**	**18 months group**	**6 months group**	**Control group**
	**(n = 8)**	**(n = 12)**	**(n = 10)**
Female / Male	3 F / 5 M	6 F / 6 M	5 F / 5 M
Age	25.5 (± 3.9)	22.8 (± 2.6)	22.9 (± 2.0)

### Assessments and further training

At start a pre-test on knowledge of basic life support was given. A post-test was performed after training. The knowledge tests consisted of 10 true/false statements. Two versions of the test existed in order to let each subject have different versions before and after training (provided as Additional file 1). Half of the subjects in each group started with one version, the rest with the other, after training this was reversed. Three or 6 items (depending on version) were directly related to bystander-CPR. Scores on the test were between 0-10, scores on CPR specific part were graded between 0-100 percent correct.

Before assessment of CPR skills the subjects were presented with a short (approx. 10 min) standardized lecture on BLS, including bystander-CPR in order to give a common theoretical base. Also a 5 minute familiarization to the SimMan® full-scale simulator (Laerdal Medical) and the environment (in particular how to call for help), was included.

**Figure 1:**
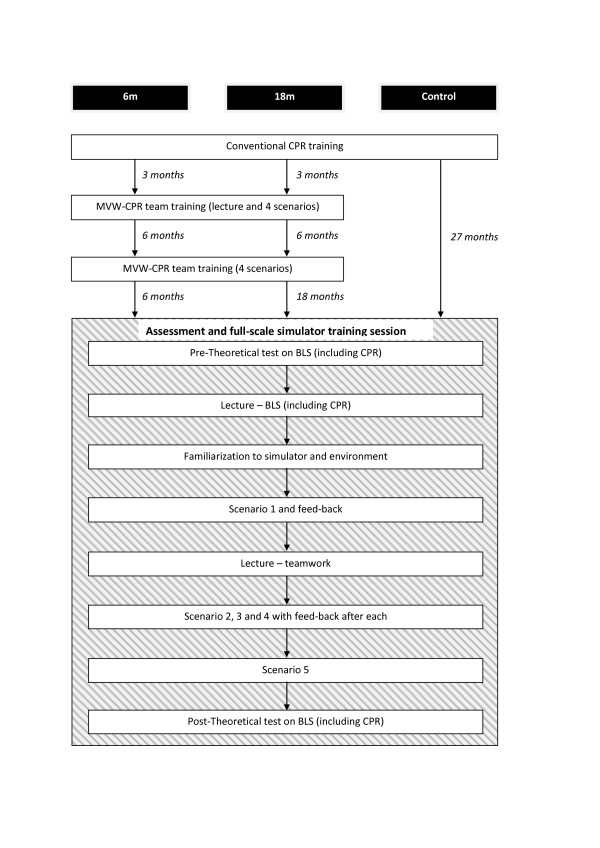
Design of the study.

The subjects were assessed and trained in teams of 2 or 3 rescuers. The first scenario was an assessment scenario. It was followed by a 20 minute standardized lecture on effective teamwork introducing the participants to so called Crew Resource Management (CRM) principles [29]. Thereafter training scenarios ensued during which two teams took turns in performing and observing. All over each team trained in 3 scenarios. In two of the training scenarios start of CPR was expected as required by the guidelines. In the third training scenario start of CPR was contra-indicated. The fifth and last scenario was again an assessment scenario. Figure 1 summarizes the design of the study.

During the scenarios the teams had to assess and resuscitate a victim by following the bystander-CPR guidelines (2005 version) [30]. At approximately 7 minutes after the yell for help the scenario was ended by help of a paramedic arriving at the scene, taking over responsibilities. After each scenario feed-back was given by an instructor and, when present, by observers. The feed-back focused on the CPR guidelines as well as on aspects of teamwork.

### Analysis and statistical methods

All scenarios were video-taped for later analysis of CPR performance. This analysis was performed by a trained blinded assessor. Several measurements of performance were used: On group level time between the rescuers entering the room until start of chest compressions was calculated and no-flow time was determined. This was calculated as the total time that elapsed between the cycles of chest compression divided by the number of cycles performed minus 1. Furthermore adherence to the CPR guidelines was scored (provided as Additional file 2). On individual level the frequency of chest compressions was calculated. Friedman repeated measures analysis of variance on ranks was used for time dependent non-parametric data. Statistical comparisons, before-after, were made by use of the Wilcoxon signed rank test. Comparisons between groups were made by ANOVA on ranks with pairwise comparisons using Dunn’s method. The test on CPR-specific knowledge was performed as a post-hoc analysis. For nominal data Chi square test was used. The significance level was set at *P* < 0.05. As P values are not adjusted for multiple testing, they have to be considered as descriptive. The calculations were performed using SigmaStat version 3.5 (Systat Software Inc, Point Richmond, CA, USA). Data are presented as mean [±SD].

## Results

### Knowledge

Table 2 describes the test scores for all groups before and after training. Total test values are presented as absolute numbers, whereas the scores on CPR-specific questions are presented as % since the two versions contained different numbers of these.

**Table 2:** Mean results on theoretical test

	**18 m**	**6 m**	**Control**	
	**(n = 8)**	**(n = 12)**	**(n = 10)**	
Total score pre-training	7.0 (±1.5)	7.7 (±0.9)	6.4 (±1.8)	18 m/6 m p = n.s.
				18 m/Control p = n.s.
				6 m/Control p = n.s.
Total score post-training	9.0 (±0.8)	8.2 (±1.0)	7.8 (±0.6)	18 m/6 m p = n.s.
				***18 m/Control p < 0.05***
				6 m/Control p = n.s.
Change pre-post	p = n.s.	p = n.s.	***p = 0.031***	
Score on CPR pre-training	73 (±23)%	93 (±11)%	65 (±28)%	18 m/6 m p = n.s.
				18 m/Control p = n.s.
				***6 m/Control p < 0.05***
Score on CPR post-training	92 (±15)%	100 (±0)%	92 (±9)%	18 m/6 m p = n.s.
				18 m/Control p = n.s.
				6 m/Control p = n.s.
Change pre-post	p = n.s.	p = n.s.	p = n.s.	

The change of overall knowledge and CPR-specific knowledge in the entire study group changed from 7.1 (±1.5) to 8.3 (±0.9) (p < 0.001) and 78% (±24%) to 95% (±10%) (p < 0.001), respectively.

### Performance

Time from subjects entering the room until start of chest compressions did not differ between groups (during first scenario: 18 months group 23 (±11) seconds, 6 months group 21 (±7) seconds and control group 24 (±13) seconds respectively) or change significantly over time. The no-flow time (time after start of CPR, where no chest compressions were administered) did not differ significantly between groups (during first scenario: 18 months group 7.9 (±2.0) seconds, 6 months group 9.4 (±2.5) seconds and control group 7.8 (±1.4) respectively, during the last scenario: 18 months group 7.5 (±0.8) seconds, 6 months group 6.8 (±1.0) and control group 6.9 (±1.8) seconds respectively). During video analysis it was evident that this variable was rather complex; if for example only one rescue breath was given, or if they were given to rapidly the no-flow time would be shorter, but at the same time the guidelines were violated.

**Figure 2:**
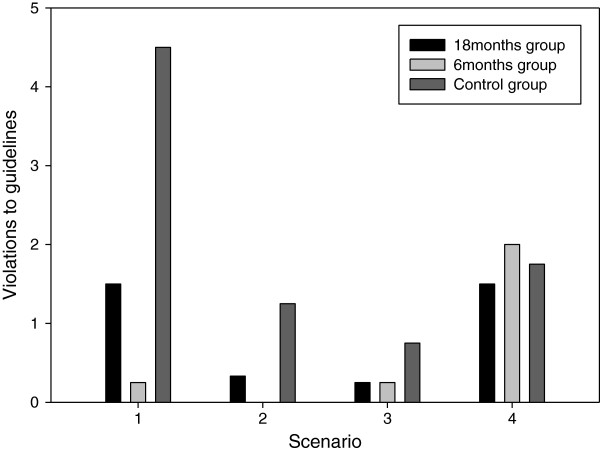
Mean number of violations to the bystander CPR guidelines in each scenario where CPR was performed.

There was a clear difference in the first scenario regarding adherence to the CPR guidelines (Figure 2). This included performing the actions in a non-prescribed order, excluding diagnostic steps, stopping to check for pulses and not performing 30:2 cycles stated by the CPR guidelines. This difference was not detectable during any of the following scenarios. We also noticed a tendency for less compliance with the guidelines during the last scenario for all groups.

**Figure 3:**
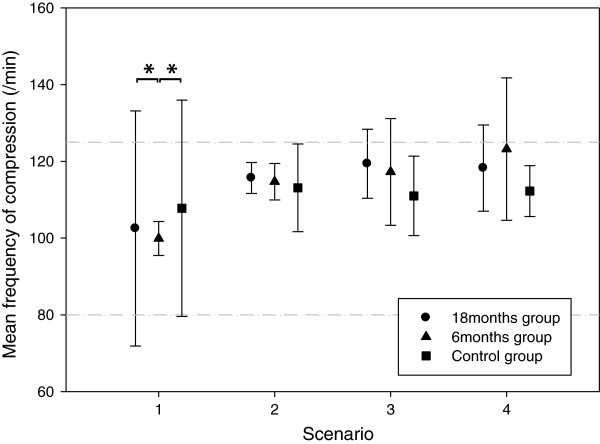
Mean frequency during cycles of chest compressions are displayed for each group in the separate scenarios.

There was also a significant difference in the number of chest compression cycles that did not agree with frequencies stipulated by the CPR guidelines during the first scenario. In the 18 months group 44 (±49)% of the compression cycles occurred at frequencies of less than 80 /min or more than 125 /min. For the 6 months and control group these values were 0% and 54 (±44)% respectively. The difference between the control group and the 6 months group was statistically significant (p < 0.001), as was the difference between the 18 months group and the 6 months group (p < 0.001). Overall, as training progressed, there was a tendency towards increased compression rates. Also, in general, the large variation of frequencies seen in the 18 months group and the control group decreased. In particular in the 6 months group the increase over time of the compression frequency led to a large proportion of cycles with frequencies over 125 /min. In the last scenario there was significantly less incorrectly paced cycles in the control group compared to the 6 months group (Figure 3).

## Discussion

In this exploratory study we found that medical students who in addition to conventional CPR training had participated in two sessions of virtual world CPR team training with avatars, performed better when assessed in full scale CPR simulation. In particular this was reflected by both better adherence to guidelines and more correct frequency of chest compressions.

### CPR knowledge

Gaps in CPR related knowledge between the groups before training and in performance during the first scenario indicated that knowledge and behaviors trained in the virtual setting were retained to some degree in contrast to lectured knowledge where no retention could be detected. After having trained in 5 scenarios, the knowledge gap in CPR related knowledge was not detectable, and the performance gap in the last scenario had disappeared.

Evidently, the rationale for CPR training is preparing potential rescuers to actually perform CPR in real life. In this respect theoretical knowledge is useful only if applied correctly. It can be argued that theoretical and correctly applied knowledge are connected [24]. Better knowledge in theory supports correct action and might provide a scaffold for future learning. Factual knowledge may also provide basis for efficacy beliefs and create a more complete mental model of the phenomenon to promote retention of procedural knowledge.

### Time before start of CPR

During real-world CPR, time is a factor of crucial importance and was consequently recorded. As shown by the large variation, in this standardized setting, this parameter was difficult to draw any conclusions from. When assessing the recordings of the scenarios for performance, we also noted a large variation in competitiveness and acting amongst the participants which in turn might have concealed actual differences in how effective the initiation of CPR was.

### Adherence to CPR guidelines

Although, due to the small sample size in this exploratory study, statistical methods have not been used on this data, support for pre-training was found in how well the CPR algorithm, stated by the bystander CPR guidelines, was followed. Virtual world pre-training reduced uncertainty and initiatives outside the defined algorithm. Interestingly, this clear pattern was found, despite the fact that all subjects just minutes earlier had attended a standardized, brief rehearsal lecture. As was hypothesized, this indicated that actual training – although carried out several months earlier in a virtual world – would still outweigh the benefits of the lecture. Hence, probably memory of subject knowledge alone is not the key to correct CPR performance.

### Psychomotor issues

Among individual psychomotor performance measures, compression frequency and quality seem to be among the most important ones for real life positive outcome. During the first scenario there was a huge variation in frequency in the 18 months and control groups. Many of these subjects were outside the stipulated frequency range. After feed-back and recurrent training this variation was considerably smaller. Notably the subjects in the 6 months group were “right on spot”, but as training progressed, showed a drift towards too high rates. This may indicate that as training goes on the participants may get overly vigorous and that more focus might be needed on this phenomenon, alternatively support the use of technical or cognitive tools for correct psychomotor output keeping frequency of compressions correct [31-33].

Another important parameter is the “no-flow time” [34,35]. In this study it was defined as the time, after start of CPR, where no chest compressions were administered. Although calculated during all scenarios, this time did not change much during the training. The lowest no-flow time was recorded during scenarios where there were several quality issues, such as reduced quality in the rescue breaths. Therefore we did not further consider this data.

New CPR training methods have been developed and refined as reactions to several problems in conventional CPR training. In a previous study we argued that the administered MVW-CPR cannot stand alone as means of CPR training [26]. However, as indicated in this exploratory study, the use of MVW-CPR pre-training might be one way of preparing students for conventional CPR courses. This added training may serve as cognitive support for future training and real-world tasks, and is in line with other trends in medicine [36]. Possibly MVW-CPR team training can also be used for rehearsal after other modes of CPR training, although it has not been the focus for the current study.

In this study the trainees are digital natives. Although CPR skills are important in many age groups, we believe that it would be easier to implement MVW-CPR team training in this group. The aged population, in which cardiac arrests most commonly occur, might also possibly benefit from such training, however from this study we can draw no such conclusions and results from such training may be much different than current results. For other digital native groups we expect that the positive effect of MVW-CPR team training would be comparable.

The strength of this study is its standardization regarding participants and protocol. CPR is often performed in groups [5]. Virtual world CPR team training with avatars has the advantage to address this issue. Although also assessments in this study to some extent were on group level, this introduced a weakness because group assessment carries the problem of decreasing statistical power. Further, the performance of groups is to some extent dependent on teamwork skills which can differ between different group constellations. One way of trying to reduce such influences was by adding a lecture on teamwork to the training program. Another limitation of this study is its small sample size. The study was conceived as a follow-up on subjects that had already received virtual CPR training. The limited sample was further reduced by loss of subjects. We therefore stress the exploratory nature of the study and must hence remember that the results have to be interpreted with caution. The validity of assessing CPR skills by use of full-scale simulators can also be questioned. To our knowledge no studies on this issue have been performed. On the other hand, this technique for training and assessment is already implemented for clinical training of skills and behaviors in various settings and scenarios [37].

A focus of this training study was to standardize the scenarios in order to evaluate how pre-training and subsequent full-scale training affected the participants. Full-scale simulator team training is costly and in very limited supply, and therefore cannot be seen as a realistic future alternative for larger groups in society. In this study, in the absence of real-world alternatives, full-scale simulation was mainly used as a way of creating a reasonably realistic way of assessing transfer.

Serious games for learning and training are receiving much attention. However, transfer of CPR skills trained in serious games is difficult to test in authentic CPR. Thus, this exploratory quasi-transfer study was designed to probe the potential future use for virtual worlds in emergency medicine.

## Conclusions

In this exploratory study we have demonstrated that conventionally trained medical students who in addition received multiplayer virtual world CPR team training in a serious game using avatars, showed better knowledge and performance when assessed in full scale CPR simulation. Better skills in terms of correctness were observed proximal to training. During repetitive training, already after a single scenario, these differences were greatly reduced demonstrating the steep proficiency gain of experiential learning compared to traditional lectures. The possibility to practice in a group setting during the virtual world training, as well as during scenario based full scale simulation addresses the need for group-focused training of CPR.

## Abbreviations

ANOVA: Analysis of variance; BLS: Basic life support; CPR: Cardiopulmonary resuscitation; CRM: Crew resource management; MVW: Multiplayer virtual world.

## Competing interests

The authors declare that they have no competing interests.

## Authors’ contribution

JC assisted in technology development, helped conceive the study, participated in study design and served as subject matter expert (emergency medicine), acquired and analyzed data, took part in data interpretation as well as drafted the manuscript. LH contributed substantially to study design, served as subject matter expert (psychological assessment), interpreted data and critically revised the manuscript. LFT conceived the study and participated in study design as well as interpreted data and critically revised the manuscript. All authors read and approved the final manuscript.

## Supplementary Material

Additional file 1 Knowledge quiz (A and B). Click here for file

Additional file 2 Adult basic life support: Assessment of CPR. Click here for file
