# Supplementary material for: Effects of pre-training using serious game technology on CPR performance – an exploratory quasi-experimental transfer study
Source: Scand J Trauma Resusc Emerg Med. 2012 Dec 6;20:79. doi: 10.1186/1757-7241-20-79 (PMC3546885; doi:10.1186/1757-7241-20-79)
Supplement: Additional file 1 — Knowledge quiz (A and B). [file 1757-7241-20-79-S1.doc]

# Knowledge quiz - A

The questions or statements presented are either correct of false. Please mark your answer in the box.

1. When you meet a person that is awake and seem to have difficulties to inhale you should immediately start to administer rescue breaths (mouth-to-mouth).

Correct  False

2. In order to reduce swelling of the brain it is good to put something under the head of an unconscious person when you don’t have to perform CPR.

Correct  False

3. According to present adult CPR guidelines you administer 15 chest compressions in each cycle.

Correct  False

4. When you have reached a severely ill (sudden onset) or traumatized person, you first check to see if she/he is conscious.

Correct  False

5. A reliable way to assure that a person is breathing is to look for chest movements.

Correct  False

6. When the airway is blocked and you can’t find a foreign body in the mouth or throat, the most common cause is an obstruction below the vocal cords.

Correct  False

7. A good pace for chest compressions is 60 / minute.

Correct  False

8. When performing B-CPR, a rescue breath should take about 1 second.

Correct  False

9. It is important to assure that the victim doesn’t have any circulation before starting B-CPR since there are obvious risks to damage the heart if chest compressions are administered while the heart is beating.

Correct  False

10. If you encounter a person that suddenly has turned severely ill, it is suitable to start the assessment of her/him by checking the airway, after first having checked for consciousness.

Correct  False

# Knowledge quiz - B

The questions or statements presented are either correct of false. Please mark your answer in the box.

1. According to B-CPR guidelines you should assure that the victim doesn’t have pulses before commencing CPR.

Correct  False

2. Before calling for help you should check if the victim is unconscious.

Correct  False

3. At the scene of a traffic accident you should first find out if an ambulance is on its way before attending the victims.

Correct  False

4. By cautiously turning the head to the right side and at the same time bending it forward you can often manage an occluded airway.

Correct  False

5. Heimlich’s maneuver is particularly suitable for unconscious persons.

Correct  False

6. When a person is unconscious, doesn’t move and doesn’t breathe, you should start B-CPR.

Correct  False

7. During B-CPR one should pause after 5 minutes to check if the victim has regained pulses.

Correct  False

8. If the circulation hasn’t recovered after 15 minutes, one should terminate B-CPR.

Correct  False

9. Because you lose in strength during chest compressions, it is recommended that you, if possible, relieve each other every 4th minute.

Correct  False

10. During rescue breaths one should aim for the chest to rise. If it doesn’t, current guidelines directs you to go on to chest compressions because it takes too long to try again.

Correct  False
