# Supplementary material for: Effects of pre-training using serious game technology on CPR performance – an exploratory quasi-experimental transfer study
Source: Scand J Trauma Resusc Emerg Med. 2012 Dec 6;20:79. doi: 10.1186/1757-7241-20-79 (PMC3546885; doi:10.1186/1757-7241-20-79)
Supplement: Additional file 2 — Adult basic life support: Assessment of CPR. [file 1757-7241-20-79-S2.doc]

Adult basic life support – Cardiopulmonary resuscitation

Unresponsive?

Shout for help

2 rescue breaths, 30 compressions†

30 chest compressions

Call 112*

Check if breathing normally

Open airway

Assessment of compliance to guidelines: A violation occurs every time the steps do not occur in correct order. This may involve additions, omissions or incorrect order. Furthermore a violation also occurs if the wrong number of chest compressions or rescue breaths occurs.

*According to 2005 ILCOR CPR guidelines published in 2005 International Consensus on Cardiopulmonary Resuscitation and Emergency Cardiovascular Care Science with Treatment Recommendations. Part 2: Adult basic life support. Resuscitation 2005, 67(2-3):187-201.*

* European guidelines

† Rescue breaths and chest compressions thereafter alternately continue until help arrives.
